# Vision under mesopic and scotopic illumination

**DOI:** 10.3389/fpsyg.2014.01594

**Published:** 2015-01-22

**Authors:** Andrew J. Zele, Dingcai Cao

**Affiliations:** ^1^Visual Science Laboratory, School of Optometry and Vision Science & Institute of Health and Biomedical Innovation, Queensland University of TechnologyBrisbane, QLD, Australia; ^2^Visual Perception Laboratory, Department of Ophthalmology and Visual Sciences, University of Illinois at ChicagoChicago, IL, USA

**Keywords:** vision, rods, cones, scotopic, mesopic, photopic, color, temporal

## Abstract

Evidence has accumulated that rod activation under mesopic and scotopic light levels alters visual perception and performance. Here we review the most recent developments in the measurement of rod and cone contributions to mesopic color perception and temporal processing, with a focus on data measured using a four-primary photostimulator method that independently controls rod and cone excitations. We discuss the findings in the context of rod inputs to the three primary retinogeniculate pathways to understand rod contributions to mesopic vision. Additionally, we present evidence that hue perception is possible under scotopic, pure rod-mediated conditions that involves cortical mechanisms.

## INTRODUCTION

The visual system is responsive to continual changes in the spectral, spatial, and temporal properties of the illuminant across ∼10 log units of dynamic range (Hood and Finkelstein, 1986). This is accomplished, in part, by switching operations between two photoreceptor classes in the retina, rods and cones, which have partially overlapping operating light ranges. Under high illuminations, rods are in saturation and photopic vision (Maxwell, 1860; Helmholtz, 1924; Hurvich and Jameson, 1957; Hering, 1964; DeValois and DeValois, 1993) is initiated by the outputs of three cone photoreceptor classes (L-, M-, and S-cones) with overlapping spectral sensitivities (Smith and Pokorny, 1975) to provide trichromatic color perception. With intermediate, mesopic illuminations when rods gradually become sensitive and cones are still active, there are subtle changes and a reduction in both the perceptual quality and gamut of perceivable colors (Nagel, 1924). Under dim, scotopic illuminations, only rods are active and color perception is still possible by different physiological computations than the trichromatic system (Pokorny et al., 2006, 2008; Elliott and Cao, 2013).

Photoreceptor outputs are transmitted from retina to brain for image forming vision via three major classes of retinal ganglion cells in primates that process distinct aspects of visual information (Dacey, 2000; Kaplan, 2004; Lee et al., 2010). The first class, known as parasol ganglion cells, project to the magnocellular (MC) layer of the LGN. The parasol ganglion cells display ON-center, OFF-surround antagonistic receptive field structures, with L- and M-cones contributing to both the centers and surrounds (spatial opponency; Rodieck, 1991). There are two subtypes of parasol ganglion cells based on the sign of the center response, including +(L+M) for ON-center cells and -(L+M) for OFF-center cells. The MC-pathway is believed to the physiological substrate of the luminous efficiency function (Lennie et al., 1993). The second class, known as midget ganglion cells, receives differential L- and M-cone inputs in the receptive field center and surround. There are four subtypes of midget ganglion cells, depending on the type and sign of cone input in the center, including +L/-M (ON response to L-cone input but OFF-response to M-cone input in the center), –L/+M, +M/-L, and -M/+L. The surround of midget ganglion cells, however, can receive mixed inputs from both L- and M-cones instead of only one type of cone input (Lee et al., 2012). Therefore midget ganglion cells display both “spatial opponency” and “chromatic opponency” to signal both spatial and chromatic (red–green) information. The notion that spatial and chromatic information is conveyed by two separate channels (“two-channel hypothesis” proposed by Rodieck, 1991) has now been dismissed. The midget ganglion cells project to the parvocellular (PC) layer in the LGN and mediate the “red–green” chromatic opponency signal and spatial acuity. The third class, known as small bistratified ganglion cells, has a spatially co-extensive center and surround receptive field structure that receives excitatory S-cone input and inhibitory L+M input. These cells project to the koniocellular (KC) layer of the LGN and are believed to mediate blue–yellow chromatic processing. Because the spectral signatures of the primary retinogeniculate neurons differ from human color perception (DeValois and DeValois, 1993), cortical transformations of these retinal projections (Calkins, 2004) and small populations of LGN cells with circuitry matching hue perception (Tailby et al., 2008) is necessary (Neitz and Neitz, 2008). Rod contribution to visual perception under mesopic illuminations is believed to be mediated via rod and cone inputs to the three pathways (Lee et al., 1997; Crook et al., 2009; Field et al., 2009; Cao et al., 2010), with rod signals merging into the cone pathway in the retina either through the rod–cone gap junctions or through rod bipolars and AII amacrines to cone bipolars (Daw et al., 1990; Sharpe and Stockman, 1999). This paper reviews current progress in understanding rod contributions to chromatic and temporal aspects of vision.

The original determinations of the Duplicity Theory of Vision (Schultze, 1866; von Kries, 1896; Müller, 1930; Saugstad and Saugstad, 1959; Stabell and Stabell, 2009) proposed separate and independent rod and cone functions, but the anatomical and physiological reality is that rods and cones share neural pathways in the retina (Polyak, 1941; Daw et al., 1990; Wassle et al., 1995; Sharpe and Stockman, 1999). The study of mesopic vision within a range of 3-4 log units of illumination (CIE, 1989, 1994) when there is a dual processing of rod and cone signals, is about revealing the nature of interactions between rod and cone photoreceptor signals. Between daylight and darkness (namely, dawn and dusk), as well as in many modern indoor lighting settings and most nighttime outdoor and traffic lighting environments, the visual system combines rod and cone signals and rod–cone interactions can modify perceptual experience and alter almost every aspect of visual processing, including visual detection (Buck et al., 1997; Sun et al., 2001b) and discrimination (Knight et al., 1998; Cao et al., 2008b), hue perception (Willmer, 1949; Lie, 1963; Trezona, 1970; Stabell and Stabell, 1971; Buck et al., 1998), color vision (Cao et al., 2005, 2008a; Pokorny et al., 2006), temporal vision (Kremers and Meierkord, 1999; Sun et al., 2001c; Cao et al., 2006; Zele et al., 2008; Cao and Lu, 2012; Zele et al., 2012, 2013), and spatial vision (Lange et al., 1997). Buck (2004, 2014) has comprehensively reviewed the effects of rod and cone interactions on human vision.

Determining the physiological substrates and mechanisms of rod–cone interaction, and how these give rise to the altered perceptual experience under mesopic illumination are largely unresolved problems in visual neuroscience. Historically, many estimates of sensitivity, magnitude, and timing of the interaction are limited by methodological approaches that inadvertently alter the relative excitation of rods and three cone classes in an undesirable manner with variation in the stimulus parameters. A central challenge in the study of mesopic vision is therefore to develop methodologies to measure rod and cone signal contributions separately and during rod–cone interaction.

## CLASSICAL EXPERIMENTAL METHODS FOR THE STUDY OF MESOPIC VISION

**FIGURE 1:**
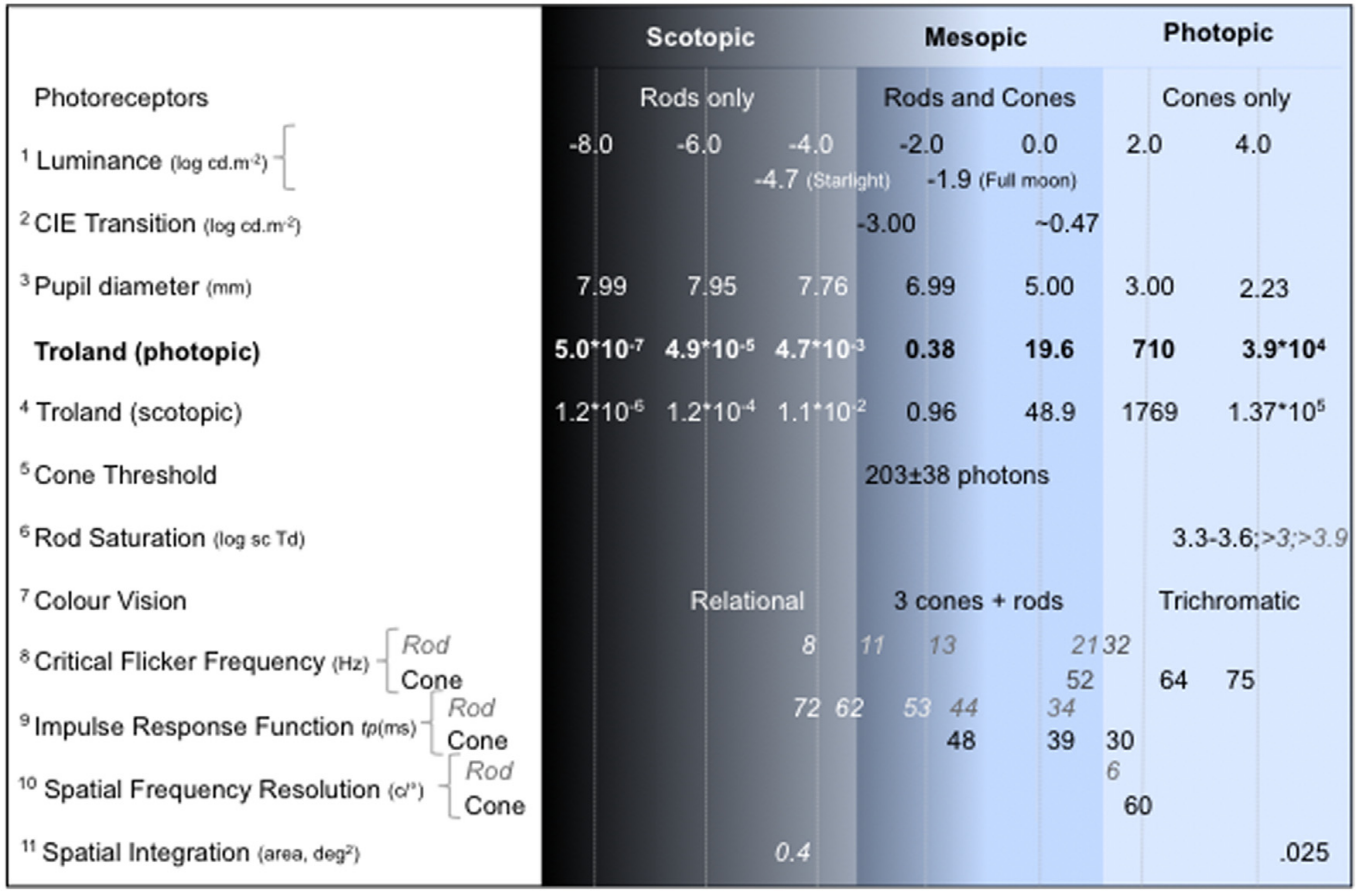
**Visual functions under scotopic, mesopic and photopic illumination.** The luminance level (log cd.m^-2^) is the reference for estimating pupil diameter using the empirical relation between pupil diameter (mm) and field luminance (Crawford, 1936; Pokorny and Smith, 1997). The reported values align approximately with the photopic retinal illuminance (photopic Troland is shown in bold) of the measurement conditions in the referenced study. When rod and cone values are both reported, *rod values are italicized*. Note that the reported values can vary with the observer, stimulus and measurement condition; see References for details of the experimental conditions: ^1^Estimate of the light level under starlight and full moon from Smith et al. (1994); ^2^CIE (1994): the transition values are complexly dependent on the viewing conditions (for example, see estimates of the rod saturation range); ^3^Crawford (1936); Pokorny and Smith (1997); ^4^Km_sc_/Km_ph_ = 2.49; ^5^Cone threshold estimated at the cornea (Koenig and Hofer, 2011); ^6^Aguilar and Stiles (1954), Shapiro (2002), Kremers et al. (2009). ^7^Pokorny et al. (2006); ^8^Rod data from Conner and MacLeod (1977); Hess and Nordby (1986), Sun et al. (2001a); cone data from de Lange (1954); Kelly (1961); ^9^Time to peak (tp ms) of the impulse response function from Cao et al. (2007); ^10^Campbell and Green (1965); Hess and Nordby (1986); ^11^Barlow (1958).

The methodologies developed to differentiate outer retina signaling are typically based on known functional differences between the rod and cone systems (**Figure 1**), and apply these methods to study rod–cone interactions arising between the stimulus area and surround (lateral interactions) or within the stimulus area (local interactions). In one method, measurements are obtained during adaptation to darkness after exposure to a bleaching light; first for the initial cone plateau adaptation phase during which only cones are sensitive, and then during the full dark-adapted phase in which both rods and cones are sensitive, but when rods are more sensitive than cones (Hecht and Hsia, 1945). As fully sensitive cones are functional during the later dark adaptation phase, rod responses can be affected by cone involvement and so there may be incomplete rod isolation. The adaptation state of rods and cones affects their relative sensitivities such that short wavelength adaptation decreases the slope of the scotopic threshold versus intensity (TvI) curve [0.72–0.78; Sharpe et al., 1992; Shapiro et al., 1996b; although S-cone signals do not appear to regulate rod sensitivity (Shapiro, 2002)] whereas L-cone excitation desensitizes the rods and steepens the TvI slope from 0.80 to 0.98 (Shapiro, 2002). The scotopic TvI function is more sensitive at higher illumination levels (Sharpe et al., 1992; Shapiro et al., 1996b; Shapiro, 2002) and rods remain active at higher illuminances (Shapiro, 2002; Kremers et al., 2009) than reported initially by Aguilar and Stiles (1954), meaning that the effect of rod intrusions may be underestimated in many experiments. Although the rod system has lower contrast sensitivity (ΔL/L = ∼0.14 vs. ∼0.015), its higher amplification enables single photon responses (Hecht et al., 1942). The visual resolution of the two systems varies by some 1200:1 between dim scotopic illuminations (∼10 min arc) and bright photopic illuminations (∼5 s arc; Hecht and Mintz, 1939). Note that thresholds for acuity and contrast sensitivity are more complex than simple TVI curves for the rod and cone systems (Barbur and Stockman, 2010). Importantly, because rod activity is optimized for low illumination and cones for high illumination, mesopic rod–cone interactions reflect signal processing at the extremes of the photoreceptor operating ranges.

Foveal and parafoveal measurements have been used to compare cone and rod function because cones are the predominant photoreceptor class in the fovea and rods are most prevalent in the parafovea (Curcio et al., 1990). This approach is limited by eccentric variations in the relative rod and cone densities, the temporal properties of rod vision (Raninen and Rovamo, 1986) and chromatic properties of cone vision (Moreland and Cruz, 1959). Moreover, rod–cone interactions may be affecting measurements and foveal and parafoveal stimuli may not truly reflect isolated cone or rod function. There is also no single eccentricity with only rod photoreceptors, unlike that for cones. The receptive fields sizes of the rod and cone system also differ. Compared to the spatial integration properties of the cone system, the rod system has larger areal summation (up to 0.4 vs. 0.025 deg^2^; Barlow, 1958), a lower peak spatial contrast sensitivity (0.5 c/° vs. ∼4.0 c/°) and cut-off frequency (∼6 c/° vs. ∼60 c/°; Campbell and Green, 1965; Hess and Nordby, 1986), and so the differential responsivities of the two systems to the spatial frequency characteristics of the stimulus (e.g., area, edges, and borders; Barbur and Stockman, 2010) must be carefully balanced within the experimental design and in the interpretation of the data.

The different spectral sensitivities of the two systems provide basis for isolating their responses. Long-wavelength adapting lights have been used to preferentially desensitize cones, but complete rod isolation is not achieved because rods and cones have roughly the same sensitivity at long wavelengths in the dark-adapted eye, and short-wavelength (S) and middle-wavelength (M) cones are not completely desensitized by long wavelength adapting lights (Crawford and Palmer, 1985). Another method is to use high temporal frequencies to bias detection to cones (Coletta and Adams, 1984). Rod system temporal integration is longer than the cone system (100 ms to ∼1s vs. ∼10-50 ms; Barlow, 1958) and both the scotopic peak temporal contrast sensitivity (5–9 Hz vs. 8–10 Hz) and maximum critical frequency is lower (20–28 Hz) than photopic vision (50–60Hz; de Lange, 1954; Conner and MacLeod, 1977; Sun et al., 2001a). Rod and cone temporal sensitivities may be more similar, however, depending on the mesopic adaptation level, spectral properties of the illuminant and stimulus eccentricity, and complete cone isolation may not be achieved. Another approach is to take advantage of the Stiles–Crawford effect and focus the light from the test field near the edge of a fully dilated pupil, which reduces the quantal efficiency of the cones, but not rods (Aguilar and Stiles, 1954). Taken together, the aim is to develop a method that controls for the distinct functional response properties of the rod and cone systems, in addition to differences in their retinal distributions, spectral sensitivities, sensitivity regulation, retinogeniculate, and higher order processing, factors that underlie the challenges and complexities encountered in the study of mesopic visual function.

## INDEPENDENT CONTROL OF ROD AND CONE SIGNALING WITH A FOUR-PRIMARY PHOTOSTIMULATING METHOD

Standard signal generators with three-primary lights are sufficient to achieve independent control of rods and two cone photoreceptor classes in dichromatic observers (Knoblauch, 1995; Kremers and Meierkord, 1999), but not the rods and three cone photoreceptors in trichromats to study rod–cone interactions. To do this, isoscotopic lines can define the combination of three primary lights with a constant scotopic luminance (fixed level of rod activity) within the domain of combinations with a constant photopic luminance, but even so it is not possible to control both scotopic and photopic luminance using three primaries in trichromats (Shapiro et al., 1996a). To achieve independent control, the number of primary lights must be no less than the number of active photoreceptors.

The four-primary method overcomes limitations of traditional methods to allow independent control of the excitation of the rod and three cone photoreceptors at the same chromaticity, adaptation level and retinal locus (Sun et al., 2001a; Pokorny et al., 2004). The theoretical basis for four-primary photostimulating methodology is silent substitution, as defined by Shapiro et al. (1996a). Effectively, the four-primary wavelengths are carefully chosen to maintain the excitation levels of some photoreceptor classes while varying the excitation of specific photoreceptor classes using silent substitution (Estévez and Spekreijse, 1982).

Considering silent substitution in color matching provides an example of independent control of rod and cones experimentally. In color matching, the chromaticity of an equal-energy spectrum light can be metamerically matched using a combination of three primary lights of different wavelengths (e.g., 460, 516, and 660 nm). The same chromaticity can also be matched using a different set of primary lights (e.g., 460, 558, and 660 nm). When a metameric match is determined for each of the sets of primaries, the L-, M-, and S-cone excitations will be equal for both matches. In this example, the two stimuli differ in only one primary (either 516 or 558 nm) while the other two primaries (460 and 660 nm) are the same in both stimuli. Since rods are more sensitive to 516 nm light than 558 nm light, switching these two sets of metameric primaries over time produces rod modulation while maintaining constant cone excitations. A similar approach can be applied to isolate L-, M-, or S-cones.

The four-primary photostimulating method offers several advantages in the study of mesopic vision. First, it can modulate one photoreceptor class while keeping the excitations of the other three photoreceptors constant, thereby allowing analysis of the contribution of only one photoreceptor to visual perception. Second, the four-primary colorimeter can maintain the same mean cone chromaticity and luminance level, while changing rod or cone excitations. Third, since the four-primary method can modulate L-, M-, and S-cone excitation independently, the independent control of postreceptoral signals, defined in MacLeod and Boynton cone chromaticity space as L/(L+M) and S/(L+M), can be easily achieved, which is not the case with other methodologies.

The four-primary photostimulating method is a better method for studying mesopic vision because direct measurements of isolated rod and cone functions and their interactions can be achieved, whereas other methods infer rod and cone functions from a comparison of measurements obtained under different conditions. Finally, the colorimeter calibration process can compensate for individual differences in pre-receptoral filtering (e.g., lens and macular pigment) to reduce errors associated with absorption of the primary lights by these filters at the plane of the retina (Pokorny et al., 2004).

## NEURAL PATHWAYS RELATED TO MESOPIC VISION

Since mesopic vision is a transitional stage between *photopic* and *scotopic* vision, it would be expected that both rod and cone signals would be sent to the cortex in mesopic conditions. The neural circuitry of the retina has been shown to allow both cone and rod signals to be transmitted to the pathways that carry information to the lateral geniculate nuclei (LGN) and then to the cortex. Physiological studies have demonstrated that, in addition to cone input, rods contribute to all three major retinogeniculate pathways. At mesopic and scotopic illuminations, physiological recordings from macaque retina indicate that parasol cells of the MC-pathway are the primary transmitter of rod signals (Gouras and Link, 1966; Virsu and Lee, 1983; Virsu et al., 1987; Purpura et al., 1988; Lee et al., 1997; Cao et al., 2010), with evidence for rod and cone signaling via small bistratified ganglion cells of the KC-pathway (Crook et al., 2009; Field et al., 2009) and midget ganglion cells of the PC-pathway (Grünert, 1997; Lee et al., 1997; Dunn et al., 2007). The analysis of natural image statistics also indicates that rods provide input to all of the three major pathways (Barrionuevo and Cao, 2014). The sharing of neural pathways allows for rod and cone signal interactions in visual system processing and provides the neural basis for rod contribution to color vision.

**FIGURE 2:**
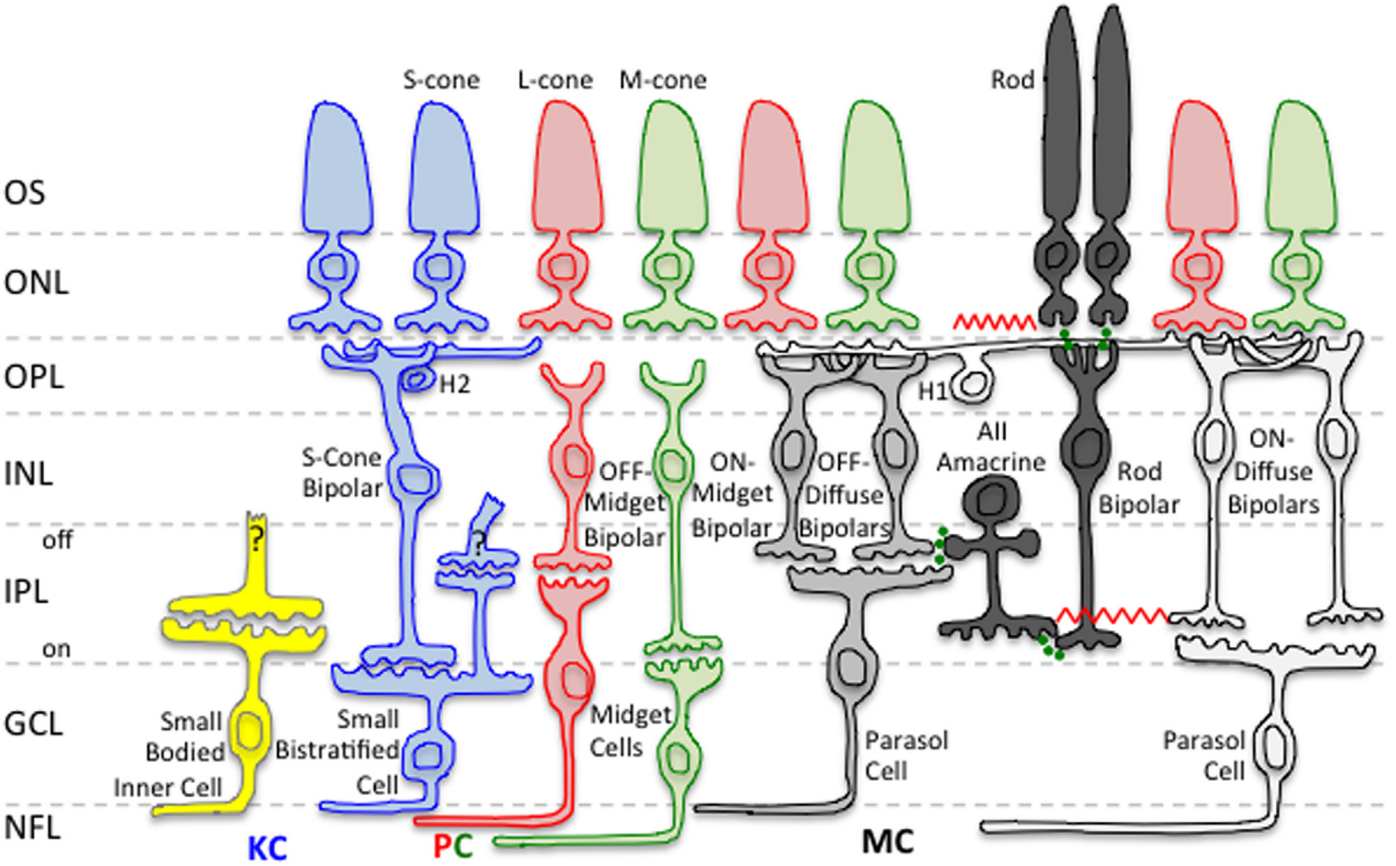
**Neural pathways related to mesopic vision.** Rod signals input to all three primary retinogeniculate pathways, namely the magnocellular (MC), parvocellular (PC), and koniocellular (KC) pathways. Only rod inputs to the MC pathway are shown in this schematic. Green circles indicate chemical synapses. The red zig–zags indicate electrical synapses. Retinal layers are indicated on the left: OS, Outer Segment; ONL, Outer Nuclear Layer; OPL, Outer Plexiform Layer; INL, Inner Nuclear Layer; IPL, Inner Plexiform Layer; GCL, Ganglion Cell Layer; NFL, Nerve Fiber Layer.

**Figure 2** shows a schematic of the two primary retinal rod pathways conveying visual information to the MC-, PC-, and KC-pathway ganglion cells that mediate different aspects of visual perception. The *rod bipolar pathway* (rods → rod bipolars → AII amacrine cells → ON/OFF cone bipolars) is a slower pathway that has been hypothesized to be active at scotopic light levels (Verweij et al., 1999). The *rod–cone gap junction pathway* (rods → cones → ON/OFF cone bipolars) is a faster pathway that has been hypothesized to be active at scotopic and mesopic light levels (Schneeweis and Schnapf, 1995; Verweij et al., 1999; Hornstein et al., 2005). However, studies have shown that, at certain light levels, both retinal rod pathways are active simultaneously in mesopic vision (Sharpe et al., 1989; Stockman et al., 1991). Therefore, the visual system has potentially a transitional stage from the *rod bipolar pathway* to the *rod–cone gap junction pathway*. The transitional range during which both retinal rod pathways are potentially functioning is thought to occur at high scotopic and low mesopic light levels.

## ROD CONTRIBUTIONS TO MESOPIC COLOR VISION

When both rods and cones are operational, rods influence all aspects of color vision (Lythgoe, 1931; Gilbert, 1950; Trezona, 1970; Stabell and Stabell, 1975a,b, 1976, 1979, 1994; Smith and Pokorny, 1977; Montag and Boynton, 1987; Buck, 1997; Cao et al., 2005). In trichromatic observers, large field and peripherally viewed lights stimulate four different photoreceptor classes at mesopic light levels and as light level decreases, large field color matches made with three primaries do not obey Grassman’s laws (Shapiro et al., 1994). Conversely, the dichromatic retina behaves similarly to the trichromatic retina with large or peripherally viewed mesopic stimuli when rods operate as the third photoreceptor class (Smith and Pokorny, 1977). Rods have been consistently shown to enhance brightness (Ikeda and Shimozono, 1981; Benimoff et al., 1982), produce brightness contrast (induction) in a central cone detected test field (Sun et al., 2001b), decrease saturation of spectral lights (Lythgoe, 1931; Gilbert, 1950; Buck et al., 1998), and improve discrimination at long-wavelengths (Stabell and Stabell, 1977). On the FM-100 hue test, rod intrusion causes discrimination loss and increased errors on the tritan axis (Knight et al., 1998). The degradation in cone chromatic discrimination that occurs in the presence of rod activity was attributed initially to rods weakening the cone signal to produce a desaturation effect (Lythgoe, 1931; Gilbert, 1950).

**FIGURE 3:**
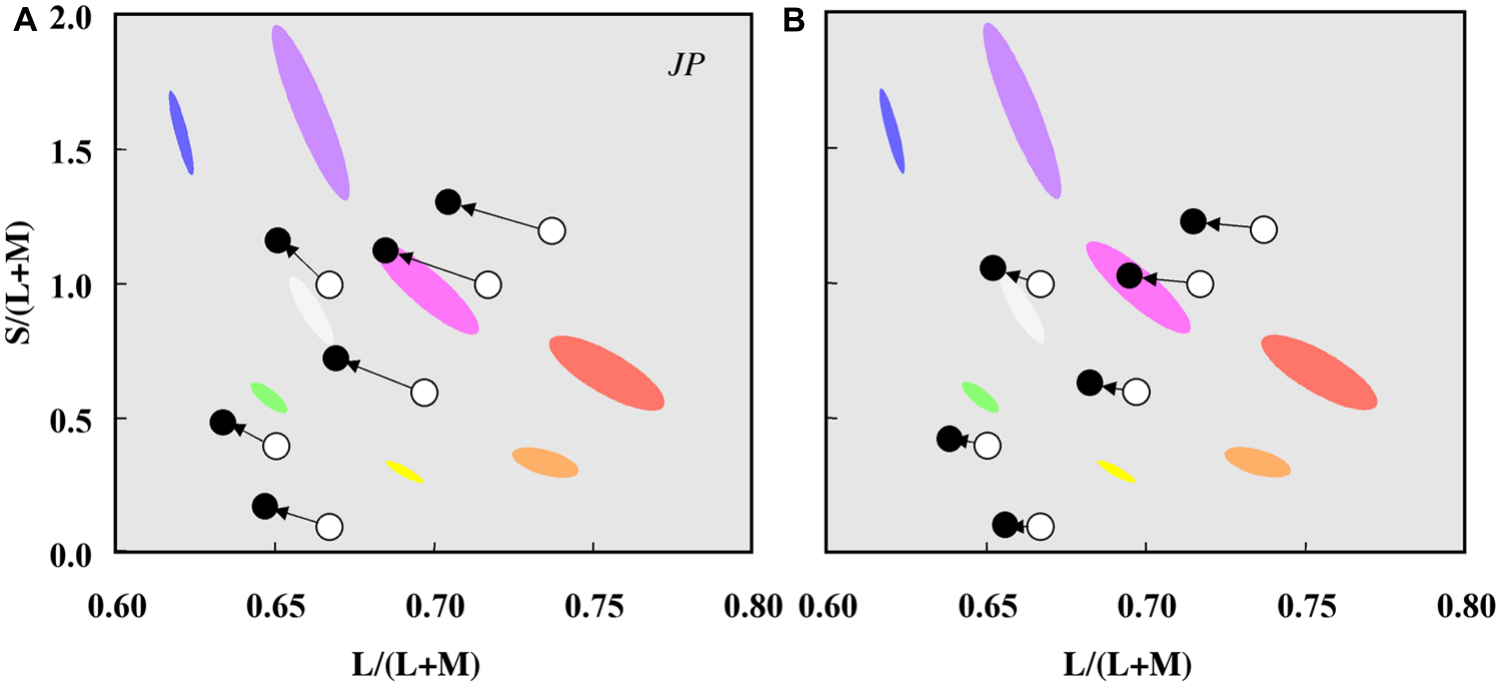
**Chromaticity shifts in the color appearance of cone signals due to rod excitation.** Data for one observer (JP) plotted in a relative cone Troland space at 2 photopic Td **(A)** and 10 photopic Td **(B)**. The arrows indicate the effect of increased rod excitation on the direction of the color shift from the stimulus chromaticities (unfilled circles) to the matching chromaticities (filled circles). Ellipses show the chromaticities of eight non-dark appearing basic colors. Adapted from Cao et al. (2005).

Color percepts associated with rod activations have been studied using unique hue measurements and hue scaling (Nerger et al., 1995; Buck et al., 1998; Nerger et al., 2003), color matching at low light levels (Trezona, 1970), scotopic color contrast (Willmer, 1949; Stabell and Stabell, 1994; Buck, 1997), by comparing measurements in foveal and parafoveal retinal locations and between dark-adapted and cone-plateau conditions (Ambler, 1974; Nagy and Doyal, 1993) and under full moon-light (Smith et al., 1994). Reports indicate that the rod percept is bluish, with evidence for multiple hue percepts (e.g., Nagel, 1924; Middleton and Mayo, 1952; Ambler, 1974; Smith et al., 1994; Stabell and Stabell, 1994; Buck et al., 1998; Nerger et al., 1998; Buck, 2001; Ishida, 2002; Shin et al., 2004). Rod activity also causes a shift (or bias) in perceived hue as demonstrated by Buck et al. (1998, 2006, 2008, 2012), Buck (2001), Knight and Buck (2002), Thomas and Buck (2004), Buck and DeLawyer (2014), and Foote and Buck (2014) in a comprehensive series of investigations that quantified three predominant hue shifts (1) the shift of unique yellow to longer wavelengths to enhance the red–green balance toward green (rod green bias), (2) the shift of unique blue to longer wavelengths (rod red bias), and (3) the shift of unique green to longer wavelengths to enhance the blue–yellow balance toward blue (rod blue bias). The critical area up to which there are no further perceived changes in hue or saturation at a given eccentricity, increases with rod activity (Pitts et al., 2005; Troup et al., 2005; Volbrecht et al., 2009). These experimental designs, however, do not yield results easily interpretable in terms of the underlying physiological mechanisms, and may be methodology dependent; for a discussion see Volbrecht et al. (2010) and Buck (2014). One reason is that a single hue sensation may not be associated with a given cone class (Knoblauch and Shevell, 2001). The most unambiguous approach is to measure the appearance of a rod signals in terms of cone activation at the same retinal location under the same adaptation conditions as achieved with a four-primary colorimeter. This negates the problems associated with a change in rod–cone excitation resulting from differences in a retinal eccentricity, illumination, or stimulus wavelength. When the four photoreceptor excitations are independently controlled (Pokorny et al., 2004), it was demonstrated that rod signals cause chromaticity shifts in directions other than toward white, demonstrating that rod activity does not lead to a purely weakened cone signal (Cao et al., 2005; **Figure 3**).

To characterize the color of rod signaling in terms of cone excitations [L/(L+M), S/(L+M), (L+M)], Cao et al. (2005, 2008a,b) used a four-primary photostimulator (Pokorny et al., 2004) and developed a perceptual matching technique that equates rod percepts with cone percepts. Because post-receptoral pathways have no information about the photoreceptor class (rod or cone) initiating the signal, rod percepts matched to cone-mediated percepts can be linked to PC-, MC-, and KC- pathway signaling. With this methodology, an incremental change in rod excitation generates a blue–greenish percept, equivalent to a decrease in L/(L+M) excitation, and increases in both S/(L+M) and (L+M) excitation (Cao et al., 2005, 2008a,b). Conversely, a decremental change in the rod excitation generates a reddish percept, equivalent to an increase in L/(L+M) excitation, a decrease in (L+M) excitation and little or no change in S/(L+M) (Cao et al., 2008b).

**FIGURE 4:**
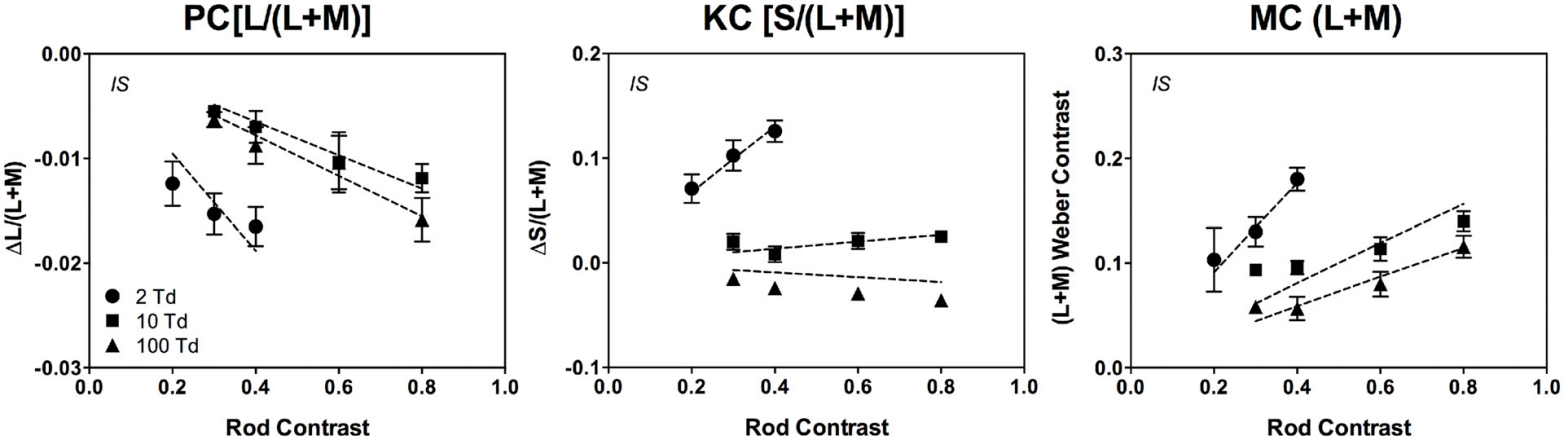
**The cone contrasts that perceptually match a rod signal are linear as a function of rod contrast.** The data were measured at 2, 10, and 100 Td (observer IS). The dashed lines are fits based on a physiologically plausible model. Adapted from Cao et al. (2008a).

Rod contributions to mesopic color perception involve differential rod signal weightings in the PC-, MC- and KC-pathways as a function of illumination level and rod contrast. The relationship between the incremental rod contrast signal (up to 80% rod contrast) and the level of PC-, MC-, and KC-pathway excitation is approximately linear (Cao et al., 2008a), consistent with physiological recordings from primate PC cells showing a linear relationship with cone contrast at all light levels, and MC cells showing a linear response at light levels less than 30 Td (Purpura et al., 1988). The rod signal strength decreases with increasing retinal illuminance in a non-linear pattern across pathways (**Figure 4**; Cao et al., 2008a) that can be described by a physiologically plausible model based on primate retinal ganglion cell responses (Smith et al., 2000; Zele et al., 2006; Cao et al., 2008a,c) with rod contributions to the cone pathways via rod–cone gap junctions at mesopic levels (Cao et al., 2008a).

In addition to affecting the perceived hue, brightness, and saturation, rod activity can alter cone-mediated chromatic discrimination (Stabell and Stabell, 1977; Nagy and Doyal, 1993; Knight et al., 1998, 2001; Cao et al., 2008b; Shepherd and Wyatt, 2008; Volbrecht et al., 2011). In comparison to measurements under photopic illumination, chromatic sensitivity measured under mesopic illuminations is differentially altered in the areas of the protan, deutan, and tritan confusion lines, with the greatest sensitivity loss near the tritan axis, but in general, the magnitude of the rod intrusion is small when measured with luminance contrast masking techniques (Walkey et al., 2001). When cone-mediated chromatic discrimination is affected by rod activity, it causes asymmetric changes for conditions where L-cone relative to M-cone excitation increases (L/M increment) and S-cone excitation decreases (S-decrement), without altering discrimination for other cone excitations (Cao et al., 2008b). Rod incremental signals degrade chromatic discrimination and rod decremental signals improve chromatic discrimination, with rod activity causing a shift in the ellipse origin and a change in the length of the minor axes (Cao et al., 2008b). Rod and cone signals combine differently in determining chromatic discrimination for different post-receptoral luminance and chromatic pathways such that the effects of rod pedestals are similar to chromatic L/(L+M) cone pedestals for L/M increment discrimination (rod contribution to the inferred PC pathway), but similar to luminance (L+M) cone pedestals for S-decrement discrimination (rod contribution to the inferred MC-pathway; Cao et al., 2008b).

## ROD CONTRIBUTIONS TO SCOTOPIC COLOR VISION

Textbook descriptions of rod contributions to color vision often state that rods signal only achromatic percepts, yet vision in twilight illumination is not always colorless (e.g., Nagel, 1924; Willmer, 1949; Thomson and Trezona, 1951; Middleton and Mayo, 1952; Lie, 1963; Ambler, 1974; Smith et al., 1994; Stabell and Stabell, 1994; Nerger et al., 1998; Buck, 2001; Ishida, 2002; Shin et al., 2004). The viewing conditions used in these studies, however, may not exclusively involve rods. Nagel (1924) noted that for illumination levels below cone threshold, many observers perceive short-wavelength reflective paper samples as blue, although he suggested that such observations do not contradict the notion of color blindness under scotopic light levels, but rather the impossibility of discriminating colors as qualities that are different from one another. Recent evidence in trichromats and dichromats brings this view into question (Pokorny et al., 2006, 2008).

With photopic illumination, cones dominate vision; the rod system is in saturation for all but the longest visible wavelengths, and their contribution to visual perception is minimal as rods in saturation do not signal stimulus change. Rod–cone coupling can, however, extend the range of rod signaling (Hornstein et al., 2005; Seeliger et al., 2011), regulate light adaptation (Cameron and Lucas, 2009) and the coupling strength may be controlled by a retinal circadian clock which increases cone receptive field size and slow the kinetics of the cone response at nighttime (Ribelayga et al., 2008). At high illuminations, rods make significant contributions to non-image forming functions via the melanopsin pathway to the circadian system (Altimus et al., 2008) and the pupil control pathway (for review Feigl and Zele, 2014). With reductions in light level from daylight to twilight, both rods and cones contribute to visual perception and further reductions lead to a selective loss in S-cone sensitivity and a progressive increase in rod sensitivity (Brown, 1951; Verriest, 1963; Walkey et al., 2001). Whilst L- and M-cones remain active, rods and L-cones primarily mediate percepts since rods are more sensitive than M-cones to mid- and short-wavelength light under twilight illumination (Pokorny et al., 2006). As rods gradually become dominant during dark-adaptation, the peak of visual sensitivity shifts toward shorter wavelengths so that objects predominantly reflecting mid- and short-wavelength light appear relatively brighter than objects reflecting long-wavelength light (Purkinje, 1825). For wavelengths greater than 650 nm, the photochromatic interval approaches zero and the rods and cones have about equal dark-adapted thresholds (Hecht and Hsia, 1945; Wald, 1945). Thus, with reductions in light level and long-wavelength stimuli, there is no situation where rods alone merdiate vision. With progressively shorter wavelengths, however, rod sensitivity increases by a factor of 1000 or greater than cones in the mid- and short-wavelength regions of the visible spectrum (Kohlrausch, 1931).

McCann and Benton (1969) observed multi-colored percepts in complex scenes illuminated with a red appearing light (656 nm) set just above L-cone threshold and superimposed with a monochromatic light (546 or 450 nm) set below cone threshold (scotopic). Such percepts were also present with Mondrian patterns (McCann, 1972). The rod contribution to this effect was determined by setting the threshold level for perception of faint blue–green, red, or yellow hues to each of 10 monochromatic lights (420–600 nm) in the presence of the 656 nm light (set just above L-cone threshold); the scotopic luminosity function matched the threshold levels of the 10 monochromatic lights (McKee et al., 1977). When the 656 nm light was increased by 1.2 log units and each of the 10 monochromatic lights were re-adjusted to find a criterion called the optimum color (that was neither too blue–green nor too red), the observers reported a wide range of hue percepts and the data again matched the scotopic luminosity function, indicating that the optimum color involved the same mechanisms. Finally, the criterion optimum color was similar irrespective of whether a 510 nm light was imaged in the pupil center or pupil periphery; rod function was implicated by an absence of the Stiles–Crawford effect. The Stiles–Crawford effect for cones was found only when the irradiance of the 656 nm light was further increased and the irradiance of the 510 nm light at the criterion optimum color needed to be set at a level above cone threshold (McKee et al., 1977). The percepts appear brighter, sharper, and slightly more saturated under photopic conditions. Taken together, these studies clearly demonstrate that multicolored percepts can be generated through interaction of L-cone and rod signals whereas independently, only (achromatic) lightness percepts are signaled. See McCann et al. (2004) for a review of rod and L-cone color.

**FIGURE 5:**
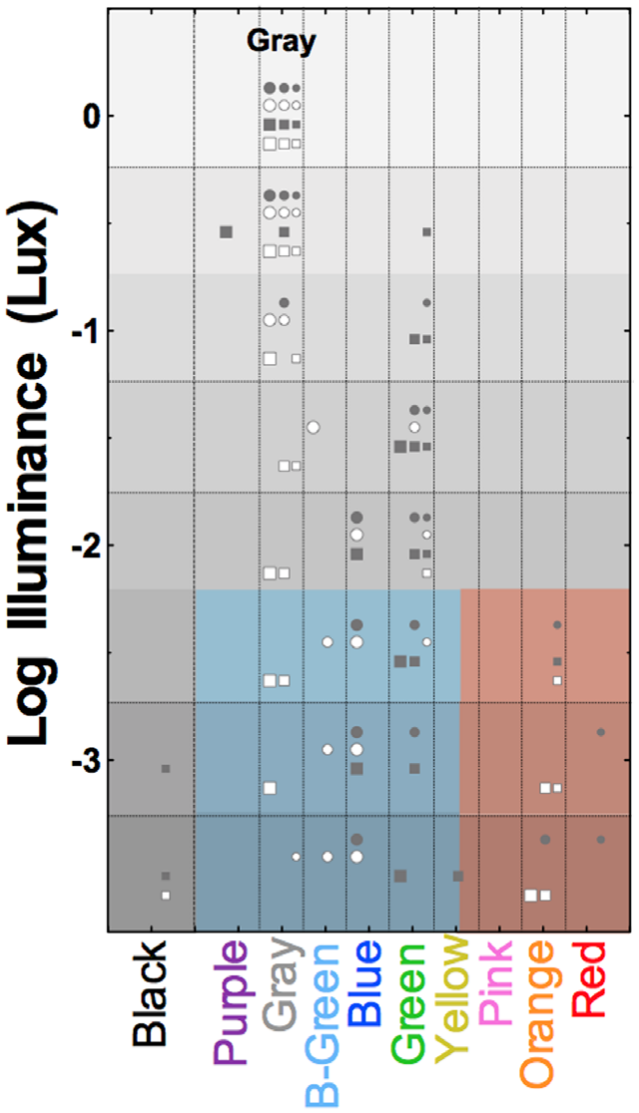
**Surface color perception under scotopic illumination reveals relational hue percepts mediated exclusively via the rod pathway.** Symbols show the reported color names from four trichromatic observers of the gray OSA-UCS color sample as a function of photopic illuminance. Symbol size refers to the lightness of the OSA-UCS sample, with lightness increasing with increasing symbol size. Light levels below about -2.25 log Lux are solely mediated by rods; the colored shaded areas demarcate the blue–green–gray and red–orange color categories used by the observers (samples below threshold were black). Adapted from Pokorny et al. (2006).

Pokorny et al. (2006) demonstrated that rods mediate variegated, scotopic hue percepts when multiple stimuli are present in the field of view. When observers are presented with an array of reflective paper samples under scotopic illumination, trichromatic participants perceive brighter appearing stimuli as blue–green–gray, and darker appearing stimuli as reddish-orange, irrespective of the photopically assigned color names. This rod color was termed *relational* because the color appearance of a paper samples changed depending on the lightness of other paper samples in view (Pokorny et al., 2006). When the samples were viewed in isolation they were perceived as blue-green, consistent with Nagel’s (1924) description. Pokorny et al. (2006) hypothesized that the visual system estimates probable color based on prior experience of viewing color in the natural environment under dim viewing conditions. **Figure 5** shows that a sample that appears gray at photopic illumination (i.e., has no chromatic information) invokes variegated color sensations at mesopic and scotopic illuminations. Such scotopic color perceptions are not restricted to trichromats; congenital dichromats have a rich color gamut under scotopic viewing conditions (Pokorny et al., 2008). Although dichromats can name color in fair agreement with color normal observers under photopic conditions (Scheibner and Boynton, 1968; Nagy and Boynton, 1979), the assigned color names under scotopic illuminations are not consistent with the scotopic lightness of the samples as were the names assigned by deuteranomalous trichromats and color normals. Pokorny et al. (2008) proposed that the limited color gamut experienced by a dichromat at photopic light levels leads to a limited association of rod color perception with objects differing in scotopic reflectance.

To understand rod color vision in complex viewing environments, Elliott and Cao (2013) investigated perceived hue in natural scene images under scotopic light levels. They showed that when a test patch had low variation in the luminance distribution and was a decrement in luminance compared to the surrounding area, reddish or orangish percepts were more likely to be reported compared to all other percepts. In contrast, when a test patch had high variation and was an increment in luminance, the probability of perceiving blue, green, or yellow hues increased. In addition, when observers had a strong, but singular daylight hue association for the test patch, color percepts were reported more often and hues appeared more saturated compared to patches with no daylight hue association. This suggests that some cortical mechanisms, which integrate experience in daylight conditions with the bottom–up rod signal processing under scotopic illumination, can modulate scotopic color perception.

## ROD–CONE INTERACTIONS IN TEMPORAL PROCESSING

Rod–cone interactions are best illustrated with stimuli that change overtime because these conditions exploit the different temporal response properties of rods and cones. While it has been demonstrated that both rods and cones contribute signals to the retinogeniculate pathways, the relative contributions of rods and cones to each of the pathways are not known. To understand how rod and cone signals are combined in different post-receptoral pathways, summation paradigms were developed to measure threshold changes as a function of the phase, contrast, and adaptation state of rods and cones (Ikeda and Urakubo, 1969; van den Berg and Spekreijse, 1977; Benimoff et al., 1982; Drum, 1982; Buck and Knight, 1994; Naarendorp et al., 1996; Kremers and Meierkord, 1999). A linear vector sum model demonstrates that a temporal combination of rod and cone signals may mediate flicker detection (MacLeod, 1972; van den Berg and Spekreijse, 1977). A non-linear combination of rod and cone signals has been shown to mediate other tasks (Buck and Knight, 1994). Because the cone pathway temporal response is faster than the rod pathway, temporal frequency dependent destructive interference between rod and cone signals causes cancelation when the signals are 180° out of phase (MacLeod, 1972; van den Berg and Spekreijse, 1977) as can occur for the putative fast and slow rod pathways (Conner, 1982; Sharpe et al., 1989; Stockman et al., 1991), but such interactions occur when the rods and cones have different sensitivities to the test stimuli and are in different states of adaptation. This is not a major factor for incremental or decremental stimuli, however, as the faster signal is processed before the slower signal (Cao et al., 2007). The type of summation also depends on the pathways mediating detection. Using a four-primary colorimetery to measure thresholds for mixed rod and L-cone (or M-cone) modulations as a function of their relative phase and frequency (2 or 10 Hz), Sun et al. (2001c) showed that probability summation occurred when rod and cone signals were mediated separately via the MC and PC pathway, and linear summation (addition or cancelation) when rods and cone signals were both mediated via the MC pathway (Sun et al., 2001c). Consistent with this observation, physiological recordings of sinusoidal stimulation of macaque parasol cells show linear summation of rod and cone signals (Cao et al., 2010).

Mesopic vision can change depending on whether signaling involves both the faster *rod–cone gap junction pathway* and the slower *rod bipolar pathway,* or when signaling shifts between these pathways. If cones are more light adapted than rods, or there is higher cone contrast, cancelation occurs at a temporal frequency where there is a 180° phase shift between the fast and slow pathways (van den Berg and Spekreijse, 1977; Conner, 1982; Sharpe et al., 1989; Stockman et al., 1991, 1995). When higher cone light adaption promotes cone signaling via the faster pathway and rod signaling via the slower pathway, cone signaling is 60–80 ms faster than rod signaling (MacLeod, 1972; van den Berg and Spekreijse, 1977; Barbur, 1982; Sharpe et al., 1989). On the contrary, when cone and rod latencies are estimated under conditions of comparable mesopic light adaptation and all photoreceptor signals are transmitted via the faster *rod–cone gap junction pathway,* the cone–rod latency difference is reduced to 8–20 ms (Sun et al., 2001b; Cao et al., 2007), in agreement with physiological estimates (Schneeweis and Schnapf, 1995; Verweij et al., 1999). The transition from the slower to the faster pathways also changes the system gain, which has been noted in rod reaction time models during the transition from high scotopic to low mesopic light levels (Cao et al., 2007).

When rods are dark-adapted in the region surrounding a cone-detected target (lateral interaction), rods suppress cone flicker detection (Lythgoe and Tansley, 1929; MacLeod, 1972; van den Berg and Spekreijse, 1977; Goldberg et al., 1983; Alexander and Fishman, 1984; Coletta and Adams, 1984; Stockman et al., 1991; Anderson and Vingrys, 2001; Cao et al., 2006; Zele and Vingrys, 2007). The mechanism and physiological substrates of lateral rod–cone interaction has been the subject of considerable debate. It was initially inferred from psychophysical studies that rods primarily interacted with L-cones when flicker sensitivity measured using stimulus conditions that caused rod excitation to vary with the wavelength of the test light (Coletta and Adams, 1984, 1985; Frumkes et al., 1988); such interpretations are not reconcilable with retinal physiology. Although early physiological reports in amphibians indicated that horizontal cells were the neural locus (Frumkes and Eysteinsson, 1988), horizontal cell inputs in primates are additive and synapse primarily with cones (Dacey et al., 1996). Using a four-primary colorimeter, it was demonstrated that lateral suppressive rod–cone interactions occur at low surround illuminances (≤0.5 Td) and are specific to receptoral (L-cone, M-cone) and postreceptoral [L+M+S] and [L+M+S+Rod] modulations containing luminance variation (Cao et al., 2006). The lateral rod–cone interaction decrease cone critical fusion frequency (CFF) by about 6 Hz (Cao et al., 2006) and reduce temporal contrast sensitivity for frequencies >6–8 Hz (Zele et al., 2008). This suppression of temporally modulated sinusoidal stimuli seems to occur in a spatial frequency range of 1–2 c/° (Lange et al., 1997). The mesopic L- and M-cone CFFs in trichromatic observers (Cao et al., 2006) are also consistent with differences in *CFF* between protanopes and deuteranopes (Smith and Pokorny, 1972; Lutze et al., 1989). That cone CFF at high mesopic light levels is suppressed by dim equiluminant surrounds (≤0.2 Td) that promote rod activity is consistent with the involvement of inhibitory signals from the AII amacrine cell directly to either cone bipolar cells or ganglion cells (Cao and Lu, 2012) and is strong in the MC-pathway (Cao et al., 2006). MC-pathway units respond vigorously to all of these modulation patterns used in the psychophysical investigation [L-, M-, L+M+S, L+M+S+Rod] (Yeh et al., 1995) and rod inputs to the retinogeniculate pathways are predominant in MC-cells (Lee et al., 1997).

Suppressive rod–cone interactions with cone isolating flicker stimuli on dim backgrounds are not significant for S-cone modulations (Cao et al., 2006) but interactions do occur when S-cones and rods are simultaneously temporally modulated (Zele et al., 2012). Interactions between rods and S-cones might be more complex, with evidence from four-primary colorimetry for linear summation of the two signals in the KC pathway which produces antagonistic, phase dependent threshold changes (Zele et al., 2012). These rod and S-cone interactions depend on the relative photoreceptor contrast ratios and a mutual, non-linear reinforcement, possibly originating at the photoreceptor level, that acts to decrease threshold (supra-additivity) with increasing contrast ratios (Zele et al., 2012). It is known from physiological studies that KC-pathway units respond vigorously to S-cone and luminance containing modulations (Yeh et al., 1995) with mesopic rod inputs to small bistratified ganglion cells in the macaque retina (Crook et al., 2009; Field et al., 2009) and with high contrast incremental stimuli in lateral geniculate nucleus of rhesus (Virsu and Lee, 1983), however, another study detected no physiologically measurable rod input to KC-ganglion units in macaque with temporal modulation (Lee et al., 1997). Physiological recording in ganglion cells have indicated that the PC-units have weak inputs from rods (Lee et al., 1997), consistent with a weak suppression of chromatic L/(L+M) signals (Cao et al., 2006). Chromatic L/(L+M) flicker detection has a different pattern of suppression and is reduced relative to that found for luminance-containing modulations, with a peak suppression at about 5 Td (Cao et al., 2006).

**FIGURE 6:**
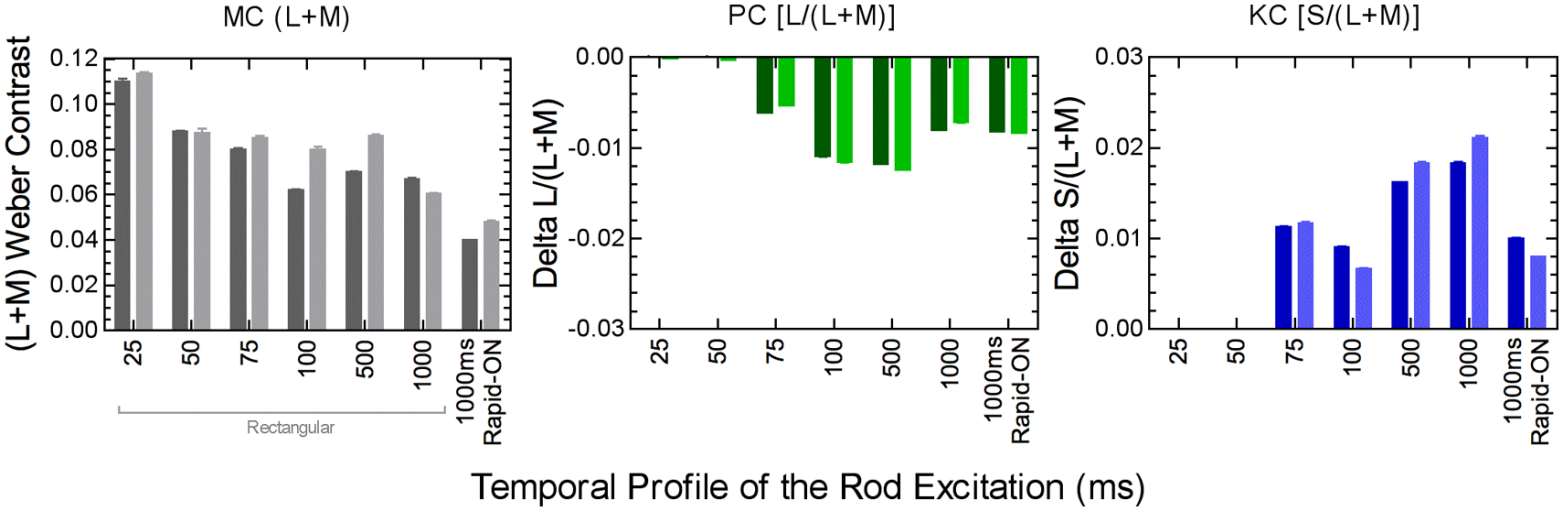
**Post-receptoral rod signaling weights in the MC, PC, and KC pathways depend on the temporal properties of the rod signal.** Data were measured using a perceptual matching paradigm at 5 photopic Td (two observers; darker and lighter columns). Adapted from Zele et al. (2013).

The relative rod contributions to the three pathways depends on the rod temporal profile. Rods produce luminance signals transmitted via the MC pathway at all measured pulse durations from 25 to 1000 ms, but only produce chromatic signals transmitted by the PC and PC pathways when the pulse duration is >75 ms (**Figure 6**; Zele et al., 2013). The implication is that the nature of the rod–cone interaction changes with the relative weighting of the rod and cone signals in the three pathways (Zele et al., 2013). The perception of motion of peripherally fixated, small circular stimuli at photopic illuminances are distorted such that the circle takes on a comet like appearance, yet long temporal responses are not typically associated with photopic vision (Barbur et al., 1986). Interestingly, the spectral response of the comet effect is consistent with rod–cone interactions, the implication being that rods in saturations can inhibit cone signaling (Barbur et al., 1986). Rod–cone interactions produce large transient sensitivity reductions at stimulus onset and offset (Buck et al., 1984; Buck, 1985; Pokorny et al., 2003; Zele and Vingrys, 2007) but the sensitivity loss due to rod–cone interactions can be less than that observed for mesopic cone–cone interactions (Zele et al., 2013). The temporal adaptation response for non-opponent cone–cone interactions is monophasic whereas opponent cone–cone interactions are biphasic (Zele et al., 2013). In contrast, different adaptation processes regulate rod and cone vision (He and MacLeod, 2000) and so an increase in the local rod adaptation level facilities rod signaling through temporal summation, pointing to some intrinsic difference in the processing of rod and cone signals in post-receptoral pathways (Zele et al., 2013).

An examination of the relationship between rod–cone interactions for stimuli that are temporally modulated (periodic) or pulsed (aperiodic) found the interactions are a general visual phenomenon affecting both periodic and aperiodic stimuli, causing the cone pathway temporal impulse response function (IRF) amplitude to decrease and the time-to peak to be delayed (Zele et al., 2008). This suppressive effect is analogous to reducing cone system contrast sensitivity and increasing the integration time (Zele et al., 2008). In the absence of an interaction, rod–cone latency differences at mesopic light levels are ∼20 ms (Schneeweis and Schnapf, 1995; Verweij et al., 1999; Sun et al., 2001c; Cao et al., 2007) and rod–cone interactions reduce this latency difference by ∼7 ms, potentially improving temporal processing under conditions where both rods and cones contribute to vision (Zele et al., 2008). Rod–cone coupling may be important for these processes.

Simple reaction times for rod or cone stimuli with various contrasts and retinal illuminances were measured (Cao et al., 2007; Zele et al., 2007) and reaction times to cone stimuli were always shorter. These measured reaction times can be modeled by rod and cone IRFs (Cao et al., 2007; Cao and Pokorny, 2010). In a separate investigation, the effects of rod–cone interaction on reaction time mediated by chromatic and luminance pathways were studied. Lateral rod–cone interactions increase cone-mediated RTs with the strongest rod–cone interactions in a dark surround (Zele et al., 2014). Reaction time has been explored as a basis for developing a real world, performance based mesopic luminous efficiency functions (He et al., 1997, 1998; Walkey et al., 2005, 2006a,b) with the assumption that reaction time is signaled via the MC pathway (He et al., 1998). Recent evidence, however, indicates there is an involvement of chromatic pathways (Walkey et al., 2006b) and rod–cone interactions alter cone mediated reaction times mediated via the chromatic and luminance pathways (Zele et al., 2014). Further developments are required to evaluate the mesopic reaction time conditions under which Abney’s law holds, as required for photopic and scotopic luminous efficiency (Lennie et al., 1993) and to ensure that the laboratory derivations of mesopic luminous efficiency are not affected by rod–cone interaction in practical real world conditions, and are robust to, or can easily accommodate, changes in relative rod and cone sensitivity that occur with changes in the viewing conditions so that its applicable in the broadest range of lighting environments.

## CONCLUSION AND FUTURE DIRECTIONS

At present there are numerous outstanding problems in the study of the dual processing of rod and cone signals. The range and impact of rod–cone interactions on human visual function and performance is only becoming known, and the subtlety and significance of these effects is becoming apparent. The cortical mechanisms for scotopic color vision are still to be defined (Pokorny et al., 2006; Elliott and Cao, 2013) and there are significant gaps in understanding the interactions between rods and S-cones for temporal processing and their roles in mesopic color perception. The quantification of the effects of rod–cone interaction on motion processing is also incomplete. To fully understand these processes under conditions best able to control for the differences between the rod and cone systems, the four-primary colorimetric method will be central. The generality of this methodology is becoming clear with new applications beyond psychophysics in the areas of physiology (Cao et al., 2010), electroretinography (Cao et al., 2011) and pupillometry (Barrionuevo et al., 2014). Combinations of these techniques will be critical for determining the physiological substrates, both in the retina and cortex, in addition to answering questions about the mechanisms of rod–cone interaction including how the relative rod and cone weights change in the post-receptoral pathways and their affects on visual function and performance.

Computational descriptions of mesopic vision derived from functional data in humans will be important for industrial applications that require optimal lighting conditions. The effects of rod–cone interaction in visual function and performance are directly applicable to many occupational environments, including transportation (i.e., aviation, maritime, rail, and road) and medicine. There are well-accepted luminosity functions for photopic and scotopic lighting conditions that are often used in science and industry, but complex nature of rod and cone contributions in mesopic illuminations means there is currently no accepted mesopic luminous efficiency function (CIE, 1989; Stockman and Sharpe, 2006). It will be important to determine general mesopic luminous efficiency functions (CIE, 1989), whether they be performance based (He et al., 1998; Walkey et al., 2006b), use minimum motion photometry (Raphael and MacLeod, 2011) or equivalent luminance (CIE, 2001), or a new method. The use of an inappropriate spectral luminous efficiency function in the mesopic region has energy efficiency and economic consequences, not to mention safety issues in, for example, lighting for nighttime transportation. Future developments in this area will include practical lighting standards for mesopic illuminations that are energy efficient and optimize visual performance. Advances in the study of mesopic vision should provide needed information for solutions to many industrial application problems. The development of a widely applicable, mesopic luminous efficiency function will be one of the most challenging problems encountered in this area of research.

We anticipate that the development of new tests for the study of rod–cone interaction in normal eyes will have great potential for translation to applied investigations (Alexander and Fishman, 1984; Arden and Hogg, 1985; Alexander et al., 1988; Falcao-Reis et al., 1991) for the development of non-invasive tests for the early detection of retinal eye disease with four-primary methodology (Feigl and Zele, 2010; Feigl et al., 2011), and for understanding the progression of retinal eye diseases that affect both rods and cones (e.g., age-related macular degeneration, diabetes, cone–rod dystrophy). New developments of clinical mesopic vision tests will be important because most acquired retinal diseases involve both the rod and cone systems. Moreover, impaired vision in the mesopic range is probably the most sensitive and earliest sign of a range of retinal diseases (Petzold and Plant, 2006). The number of complaints about disturbances in mesopic and scotopic vision after corneal refractive surgery is also increasing, indicating the importance of developing new measures of these visual disturbances (Fan-Paul et al., 2002).

In general, decision processing in perceptual detection or action tasks is cortically mediated and, therefore, the photoreceptor source of information in these tasks should not be a salient factor at the level of the cortex. In mesopic decision processing, rod and cone signals should be considered as largely interchangeable in terms of postreceptoral visual processing and decision processing for final perception (Cao and Pokorny, 2010). However, if a retinal disease preferentially affects a particular photoreceptor class, thereby affecting rod and cone contributions to the retinogeniculate pathways and rod and cone interactions, visual perception may be affected. Therefore, the study of changes in rod and cone contributions and interactions in neural pathways in diseases may be helpful in understanding the mechanisms of visual loss. Expanding the study of retinal diseases by examining visual perception under mesopic conditions may prove to be illuminating.

## AUTHOR CONTRIBUTIONS

The Authors made equal intellectual contributions to the paper and are accountable for all intellectual aspects of the work.

## Conflict of Interest Statement

The authors declare that the research was conducted in the absence of any commercial or financial relationships that could be construed as a potential conflict of interest.
